# Expression of a Malarial Hsp70 Improves Defects in Chaperone-Dependent Activities in *ssa1* Mutant Yeast

**DOI:** 10.1371/journal.pone.0020047

**Published:** 2011-05-19

**Authors:** Samantha L. Bell, Annette N. Chiang, Jeffrey L. Brodsky

**Affiliations:** Department of Biological Sciences, University of Pittsburgh, Pittsburgh, Pennsylvania, United States of America; Texas A&M University, United States of America

## Abstract

*Plasmodium falciparum* causes the most virulent form of malaria and encodes a large number of molecular chaperones. Because the parasite encounters radically different environments during its lifecycle, many members of this chaperone ensemble may be essential for *P. falciparum* survival. Therefore, *Plasmodium* chaperones represent novel therapeutic targets, but to establish the mechanism of action of any developed therapeutics, it is critical to ascertain the functions of these chaperones. To this end, we report the development of a yeast expression system for PfHsp70-1, a *P. falciparum* cytoplasmic chaperone. We found that PfHsp70-1 repairs mutant growth phenotypes in yeast strains lacking the two primary cytosolic Hsp70s, *SSA1* and *SSA2*, and in strains harboring a temperature sensitive *SSA1* allele. PfHsp70-1 also supported chaperone-dependent processes such as protein translocation and ER associated degradation, and ameliorated the toxic effects of oxidative stress. By introducing engineered forms of PfHsp70-1 into the mutant strains, we discovered that rescue requires PfHsp70-1 ATPase activity. Together, we conclude that yeast can be co-opted to rapidly uncover specific cellular activities mediated by malarial chaperones.

## Introduction

Malaria is a major worldwide health concern, infecting approximately 250 million people and killing at least one million people annually. Most of those killed by malaria are children. The disease disproportionately affects individuals in Sub-Saharan Africa, as well as in Asia, Latin America, the Middle East and parts of Europe. Malaria is caused by a protozoan infection, and *Plasmodium falciparum* is the most common and deadly species in the malarial parasite family. Unfortunately, the emergence of drug resistant malarial strains has made the disease significantly more difficult to treat. Widespread resistance to affordable and formerly effective drugs like chloroquine, which inhibits heme detoxification [1], has been well documented. Resistance to artemenisin combination therapies, which was once thought to provide a fail-safe treatment option, has now been documented along the Thai-Cambodian border [2]. Other new antimalarials are prohibitively expensive and/or are accompanied by adverse side effects [3]. Overall, it is critical that novel drug targets are identified and that the mechanism of action of putative new anti-malarials [2] are defined so that compounds with improved efficacy can be developed.

One new anti-malarial target is molecular chaperones. Molecular chaperones are defined as proteins that prevent aberrant interactions between macromolecules. As a result, this broad class of highly conserved factors prevents protein aggregation and facilitates a number of cellular processes, including protein transport, assembly, disassembly, and degradation [4]. Heat shock proteins, a group of molecular chaperones first identified by their increased expression during temperature stress [5], are classified by molecular mass. One class, the 70 kDa heat shock proteins, Hsp70s, have an N-terminal ATP-binding domain followed by a substrate-binding domain (SBD); the SBD contains a pocket to bind hydrophobic regions in substrates [6], [7]. A C-terminal lid entraps peptide substrates. In order to drive cycles of substrate binding and release, Hsp70s hydrolyze ATP: Hsp70s exhibit a low affinity and high off-rate for peptides in the ATP bound state, but they exhibit high affinity binding and a low off-rate in the ADP bound state. The release of bound ADP, and subsequent rebinding to ATP, requires at least one member of an emerging class of structurally diverse nucleotide exchange factors [8].

Hsp40s are another abundant and conserved family of molecular chaperones [9], [10]. Although Hsp40s can bind directly to hydrophobic regions of unfolded protein substrates [11], and are consequently *bona fide* molecular chaperones in isolation, they most often function as Hsp70 co-chaperones. Notably, Hsp40s stimulate the rate of ATP hydrolysis by Hsp70s and thus facilitate the binding of Hsp70s to their substrates. Therefore, the coordinated action of Hsp70s with select Hsp40 partners provides enhanced chaperone activity.

There are several reasons why heat shock protein studies in the malarial parasite are of great importance. The genome of *P. falciparum* contains sequences encoding six Hsp70s and forty-three Hsp40s [12], [13]; this large number of Hsp70s and Hsp40s in a parasite suggests that the chaperones might be crucial for survival [14], [15]. Furthermore, features of the parasite's lifecycle suggest a profound requirement for molecular chaperone function, and in particular for Hsp70s and Hsp40s. First, *P. falciparum* is carried by mosquitoes that live in ambient temperature, but the parasite then replicates in humans at 37°C and must endure temperature stresses during the episodes of fever that result from infection [16]. Second, the parasite is exposed to an oxidative stress when harbored within red blood cells [17], and Hsp70-Hsp40 function is known to mitigate the cellular toxicity that is triggered by oxidatively damaged proteins [18]. Third, during infection, *P. falciparum* establishes a unique secretory pathway within infected red blood cells. Given the many connections between Hsp70 and Hsp40 function and the secretory pathway in eukaryotes [19], these heat shock proteins might reorganize organelles within the parasite that constitute the *Plasmodium*-specific secretory pathway [20]. Fourth, the secretion of one Hsp40 homolog is required for the formation of “knobs” on infected red blood cells, which enables the parasite to survive by avoiding the splenic filtration system [21]. Finally, 15-deoxyspergualin, a compound that modulates Hsp70 activity [22]–[24], inhibits the growth of *P. falciparum* by altering protein traffic to the parasite's apicoplast [25]–[27].

Of late, there has been an increased desire to develop therapeutics that target chaperone function, in particular the function of Hsp70s [28], [29]. However, in parallel, it is vital to define the potentially unique *in vivo* activities of Hsp70s. In most cell types, this is a straight-forward procedure. Unfortunately, genetic manipulations of *P. falciparum* are cumbersome, the development of biochemical assays in this organism suffer from difficulties in preparing large amounts of starting material, and few tools are available to probe the functions of malarial gene products *in vivo*. To surmount these technical hurdles, we developed a malarial Hsp70 expression system in another eukaryote, the yeast *Saccharomyces cerevisiae*. Heterologous yeast expression systems have been used to define the etiology of numerous human diseases, and genetic manipulations are facile. Moreover, a large number of tools have been developed to dissect Hsp70 function *in vivo*. Finally, yeast expression and complementation studies have been used to explore the properties of several chaperones from other organisms (see for example [30]–[36]). The data presented in this manuscript indicate that a malarial chaperone, PfHsp70-1, facilitates many of the same Hsp70-dependent activities documented in other eukaryotes. Our study also sets-the-stage to define the activities of additional *P. falciparum*-encoded molecular chaperones [37], [38].

## Methods

### Construction of Yeast Expression Vectors for the *P. falciparum* Hsp70, PfHsp70-1, and for the Yeast Hsp70, Ssa1

Yeast strains and expression plasmids used in this study are listed in Table 1 and Table 2, respectively. Yeast expression vectors for PfHsp70-1 were constructed using the *SSA1* endogenous promoter and single copy number plasmids. The following primers were designed in order to PCR amplify the *SSA1* promoter, which includes the 500 bp untranslated region directly upstream of the *SSA1* gene: 5′–CTA GAG GAT CCA TGT CAA AAG CTG TCG GTA–3′ and 5′–CAG TTC AAG CTT TTA ATC AAC TTC TTC AAC GG–3′. SacI and BamHI restriction sites, respectively, were engineered into these primers (underlined) to facilitate subsequent cloning, and genomic DNA from a wild type yeast strain was used as a template in the PCR reaction. The amplified *SSA1* promoter was gel purified and subcloned into three *CEN/ARS* yeast expression plasmids; the *SSA1* promoter replaced the *TEF* promoter in the p416*TEF* and p414*TEF* plasmids, and the *SSA1* promoter was inserted into pRS313 between the SacI and BamHI restriction sites [39], [40]. This generated the “empty” expression vectors p416-P*_SSA1_*, p414-P*_SSA1_*, and p313-P*_SSA1_*, which were used as negative controls in our experiments. Next, to construct plasmids for the expression of Ssa1 and PfHsp70-1, plasmids containing the *SSA1* [41] or PfHsp70-1 [42] genes were digested with BamHI and HindIII, and the excised genes were ligated into the empty vectors, indicated above, using the BamHI and HindIII restriction sites. This yielded the plasmids p416-P*_SSA1_*-*SSA1*, p414-P*_SSA1_*-*SSA1*, p313-P*_SSA1_*-*SSA1*, p416-P*_SSA1_*-*PfHsp70-1*, p414-P*_SSA1_*-*PfHsp70-1*, p313-P*_SSA1_*-*PfHsp70-1*. The plasmids were verified using restriction digest and DNA sequence analysis.

**Table 1:** Yeast Strains used in this study.

Strain	Genotype	Reference or Source
BY4742	*MATα, his3Δ1, leu2Δ0, lys2Δ, ura3Δ0*	Research Genetics
*SSA1 ssa2Δ ssa3Δ ssa4Δ*	*MATα, his3-11,15, leu2-3,112, ura3-52, trp1-1, lys2, SSA1, ssa2-1::LEU2, ssa3-1::TRP1, ssa4-2::LYS2*	[47]
*ssa1-45 ssa2Δ ssa3Δ ssa4Δ*	*MATα, his3-11,15, leu2-3,112, ura3-52, trp1-1, lys2, ssa1-45, ssa2-1::LEU2, ssa3-1::TRP1, ssa4-2::LYS2*	[47]
*SSA1 SSA2*	*MATα, kar1-1, SUQ5, ade2-1, his3Δ202, leu2Δ1, trp1Δ63, ura3-52, ura2::KanMX*	[59]
*ssa1Δ ssa2Δ*	*MATα, kar1-1, SUQ5, ade2-1, his3Δ202, leu2Δ1, trp1Δ63, ura3-52, ura2::KanMX, ssa1::KanMX, ssa2::HIS3*	[59]

**Table 2:** Yeast Expression Plasmids used in this study.

Plasmid	Genotype	Reference or source
pQE30-PfHsp70	*Amp^R^, 2μ, PfHsp70-1*	[42]
p426GPD-(His)_6_-SSA1	*Amp^R^, URA3, 2μ, SSA1*	[41]
p416TEF	*Amp^R^, URA3, CEN*	[39]
p414TEF	*Amp^R^, TRP1, CEN*	[39]
pRS313	*Amp^R^, HIS3, CEN*	[40]
p416-P_SSA1_	*Amp^R^, URA3, CEN, P_SSA1_*	this study
p416-P_SSA1_-SSA1	*Amp^R^, URA3, CEN, P_SSA1_, SSA1*	this study
p416-P_SSA1_-PfHsp70-1	*Amp^R^, URA3, CEN, P_SSA1_, PfHsp70-1*	this study
p416-P_SSA1_-PfHsp70-1-G214D	*Amp^R^, URA3, CEN, P_SSA1_, PfHsp70-1-G214D*	this study
p416-P_SSA1_-PfHsp70-1-P434L	*Amp^R^, URA3, CEN, P_SSA1_, PfHsp70-1-P434L*	this study
p414-P_SSA1_	*Amp^R^, TRP1, CEN, P_SSA1_*	this study
p414-P_SSA1_-SSA1	*Amp^R^, TRP1, CEN, P_SSA1_, SSA1*	this study
p414-P_SSA1_-PfHsp70-1	*Amp^R^, TRP1, CEN, P_SSA1_, PfHsp70-1*	this study
p414-P_SSA1_-PfHsp70-1-G214D	*Amp^R^, TRP1, CEN, P_SSA1_, PfHsp70-1-G214D*	this study
p414-P_SSA1_-PfHsp70-1-P434L	*Amp^R^, TRP1, CEN, P_SSA1_, PfHsp70-1-P434L*	this study
p313-P_SSA1_	*Amp^R^, HIS3, CEN, P_SSA1_*	this study
p313-P_SSA1_-SSA1	*Amp^R^, HIS3, CEN, P_SSA1_, SSA1*	this study
p313-P_SSA1_-PfHsp70-1	*Amp^R^, HIS3, CEN, P_SSA1_, PfHsp70-1*	this study
pRS426-CFTR-HA	*Amp^R^, URA3, 2μ, CFTR-3xHA*	[46]

PfHsp70-1 mutants were generated using the QuikChange Mutagenesis kit (Stratagene). The p416-P*_SSA1_*-*PfHsp70-1* plasmid was used as a template, and the following primer pairs were used to introduce the G214D and P434L substitutions, respectively (underlined sequence indicates mutated sequence): 5′–CAT TTT AAT TTT CGA CTT AGG AGA TGG TAC ATT TGA TGT ATC ATT AT–3′ and 5′–ATA ATG ATA CAT CAA ATG TAC CAT CTC CTA AGT CGA AAA TTA AAA TG–3′; 5′–ATT GAA AGA AAC ACA ACC ATA CTA GCT AAA AAG AGT CAA ATC TTT AC–3′ and 5′–GTA AAG ATT TGA CTC TTT TTA GCT AGT ATG GTT GTG TTT CTT TCA AT–3′. The desired mutations in the resulting p416-P*_SSA1_*-*PfHsp70-1-G214D* and p416-P*_SSA1_*-*PfHsp70-1-P434L* constructs were confirmed by DNA sequence analysis, and the genes were cloned into the p414*TEF* plasmid using the SacI and XhoI restriction sites, giving plasmids p414-P*_SSA1_*-*PfHsp70-1-G214D* and p414-P*_SSA1_*-*PfHsp70-1-P434L*. These constructs were verified using restriction digest.

### Yeast Molecular and Biochemical Techniques

Lithium acetate transformation [43] was used to introduce the desired plasmids into the indicated yeast strains. Cells were grown at 26°C unless indicated otherwise, and in the indicated selective media supplemented with 2% glucose [44]. Where noted, cells were grown in the presence of cadmium chloride (Sigma), copper sulfate (Sigma), diamide (Sigma), or hydrogen peroxide (TopCare) at the indicated concentrations.

The solubility of PfHsp70-1 expressed in yeast was assessed as described [45] with minor modifications. Equivalent numbers of cells (∼10 OD_600_ units of cells) were harvested and then lysed with glass beads in 20 mM HEPES, pH 7.4, 100 mM NaCl, 20 mM MgCl_2_, and a protease inhibitor cocktail. The cleared lysates were centrifuged at 16,000 g for 15 min at 4°C and the resulting supernatant and pellet fractions were collected and analyzed by SDS-PAGE. The presence of soluble PfHsp70-1 in the supernatant was examined by western blot analysis.

Endoplasmic reticulum associated degradation (ERAD) efficiency was measured through a cycloheximide chase assay using the cystic fibrosis transmembrane conductance regulator as a substrate for this pathway [46]. In brief, cycloheximide chase analyses were performed by growing yeast cells to logarithmic phase (OD_600_ = 0.5–1.2) in selective media at 30°C. Cycloheximide was added to a final concentration of 100 µg/ml, the cells were shifted to 37°C, and aliquots of ∼1 OD_600_ units of cells were harvested at the indicated times. Proteins were isolated using trichloroacetic acid (TCA) precipitation, and western blot analysis was used to detect the indicated protein substrates. The stability of PfHsp70-1 was measured using the same method except that the appropriate antibody was used (see below).

Defects in prepro-αfactor (ppαF) translocation [41] were assessed by growing cells to logarithmic phase (OD_600_ = 0.5–1.2) at 26°C and shifting the cultures to 37°C for 15 min to induce the *ssa1* mutant phenotype [47]. The cells were then harvested (∼3 OD_600_ units of cells) and resuspended in sample buffer (80 mM Tris, pH 6.8, 2% sodium dodecyl sulfate, 0.1% bromophenol blue, 100 mM dithiotreitol, 10% glycerol). After incubating samples at 75°C for 10 min, cells were lysed with glass beads. Western blot analysis was then used to detect the untranslocated substrate, ppαF, using a specific anti- ppαF antibody that was a kind gift from R. Schekman (University of California, Berkeley).

Other antibodies used for these studies included anti-PfHsp70-1 [42], anti-Ssa1 [48], anti-Sec61 [49], anti-glucose-6-phosphate dehydrogenase (G6PDH) (Sigma), and horseradish peroxidase-conjugated anti-haemaglutinin-A (Sigma). Western blots were imaged using enhanced chemiluminescence (Pierce) and a Kodak 440CF Image Station and the resulting data were quantified using Kodak 1D software (Eastman Kodak).

## Results

### The Malarial Hsp70 homologue, PfHsp70-1, can be expressed and is stable in the yeast, *S. cerevisiae*

To examine whether the function of a malarial chaperone could be studied in another eukaryote that might be more amenable to molecular and genetic tools, we selected the *P. falciparum* Hsp70-1 protein for the following reasons. Most importantly PfHsp70-1 has been shown to be expressed in the parasite and can function as a chaperone *in vitro* [42], [50], [51]. In addition, the protein ends in an EEVD motif. This amino acid sequence is required for Hsp70 to interact with tetratricopeptide repeat domain-containing proteins, which are found in many Hsp70 co-chaperones and play an important role in cellular physiology in eukaryotes [52]. Furthermore, evidence exists for an interaction between PfHsp70-1 and a parasitic Hsp40 homolog [51], suggesting that PfHsp70-1 has the ability to interact with co-chaperones. And finally, a role for *P. falciparum* Hsp70s in protein transport has been inferred [53], suggesting that Hsp70-1 might functionally substitute for a yeast Hsp70 homolog that is similarly required for protein transport.

**Figure 1:**
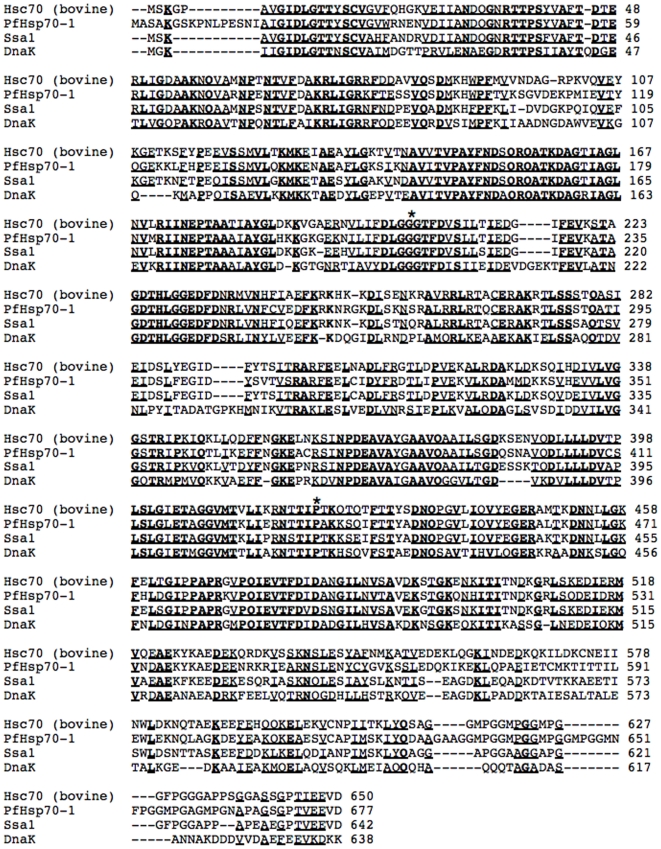
*S. cerevisiae* Hsp70, Ssa1, and *P. falciparum* Hsp70, PfHsp70-1, are >70% identical. The alignment was generated using ClustalW2 software [70]. Sequences in bold face indicate identity, and underlined sequences indicate similarity. Gaps are indicated with a dash. Asterisks denote amino acids substituted in the PfHsp70-1 mutants (see text for details).

Next, we generated yeast expression plasmids for the malarial chaperone. The PfHsp70-1 gene was subcloned and placed under the transcriptional control of a promoter for a yeast Hsp70, Ssa1, in a single copy vector. Therefore, the parasite chaperone will be under the same transcriptional control as the yeast chaperone and respond to the same cellular stress conditions. In addition, PfHsp70-1 and Ssa1 are 71% identical and 97% similar (Figure 1). The sequence identity is even higher in the ATPase domain and critical residues required for this enzymatic activity are identical between PfHsp70-1 and Ssa1; this feature is critical for the interpretation of mutagenesis studies (see below). *SSA1* encodes a constitutively expressed cytosolic Hsp70 in yeast and the gene is only mildly induced by heat shock [54]. Moreover, Ssa1 is required during the post-translational translocation of newly synthesized secreted proteins into the endoplasmic reticulum (ER) [47], [55], [56], and for the ER associated degradation (ERAD) of misfolded membrane proteins such as the cystic fibrosis transmembrane conductance regulator (CFTR) [46], [57]. Consequently, there are a number of assays that can be co-opted to assess PfHsp70-1 function. As a positive control for these studies, we also cloned *SSA1* into the same single-copy expression vector, and as a negative control, the *SSA1* promoter was cloned into the plasmid but a downstream gene was absent.

In addition to *SSA1*, *S. cerevisiae* harbors genes for the expression of three additional members of this cytosolic Hsp70 family [54]. Ssa2 is also constitutively expressed and is 98% identical to Ssa1. In contrast, Ssa3 and Ssa4 are poorly expressed but are highly stress inducible. Therefore, the function of Ssa1, or any heterologously expressed Hsp70, is best studied in the *ssa1-45* strain [47]. This strain contains a copy of *SSA1* with a point mutation that renders Ssa1 inactive at temperatures above 37°C, and both the mutant and isogenic “wild type” strains have the remaining three *SSA* genes—*SSA2*, *SSA3*, and *SSA4*—deleted to prevent their ability to complement the *ssa1-45* allele due to functional redundancy. As a second approach to probe PfHsp70-1 function, we employed an *ssa1Δssa2Δ* strain that is thermosensitive for growth and has been used to analyze the contributions of Hsp70 chaperones on various cellular processes [54], [58]–[61].

**Figure 2:**
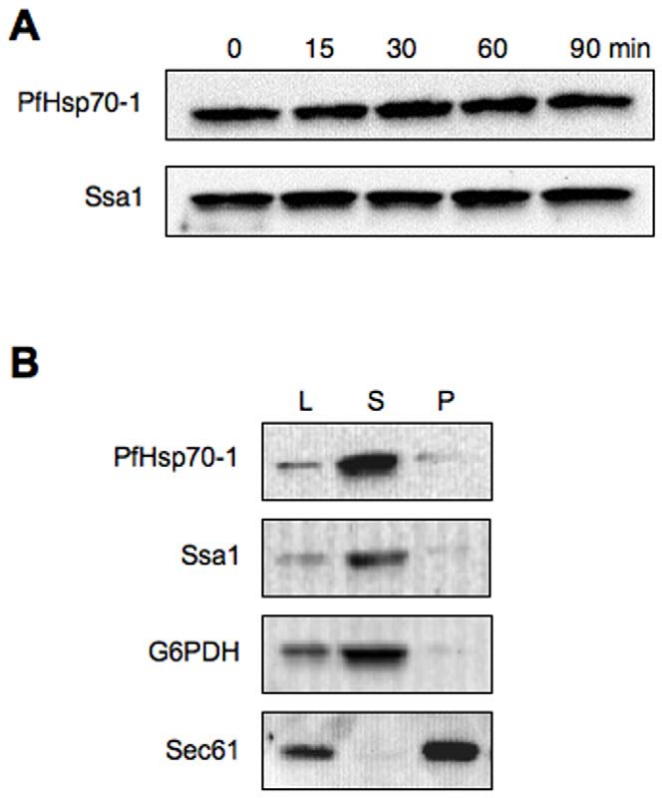
PfHsp70-1 is stable and soluble when expressed in *S. cerevisiae*. (A) A cycloheximide chase analysis was performed using a wild type yeast strain transformed with plasmid p416-P*_SSA_*_1_-*PfHsp70-1* (top) or p416-P*_SSA_*_1_- *SSA1* (bottom). The assay was performed at 30°C, and samples were taken at the indicated time points after the addition of cycloheximide. Samples were processed as described in the “Methods”. (B) Cells expressing PfHsp70-1 were lysed and processed as described in the “Methods”. Sec61 was probed as an ER membrane protein control and G6PDH was probed as a soluble (cytosolic) protein control. L: lysate; S, soluble fraction; P, pellet fraction. Please note that the lysate examined in this panel represents only a variable fraction of the total lysate that was processed to obtain the soluble and pellet samples.

We next verified the stable expression of the Pf Hsp70-1 in yeast by transforming a wild strain, BY4742 (Table 1), with the *SSA1* expression vector and the PfHsp70-1 expression vector. To examine the comparative stabilities of the two Hsp70s, a cycloheximide chase analysis was performed. Figure 2A shows that PfHsp70-1 is as stable as the endogenous yeast homolog, Ssa1. To ensure that the heterologously expressed protein is also soluble, cells expressing the proteins were lysed and cytosolic fractions were prepared. Figure 2B shows that PfHsp70-1 remains soluble when expressed in yeast.

### PfHsp70-1 partially rescues the temperature sensitivity and oxidative stress of yeast deficient in cytoplasmic Hsp70 function

**Figure 3:**
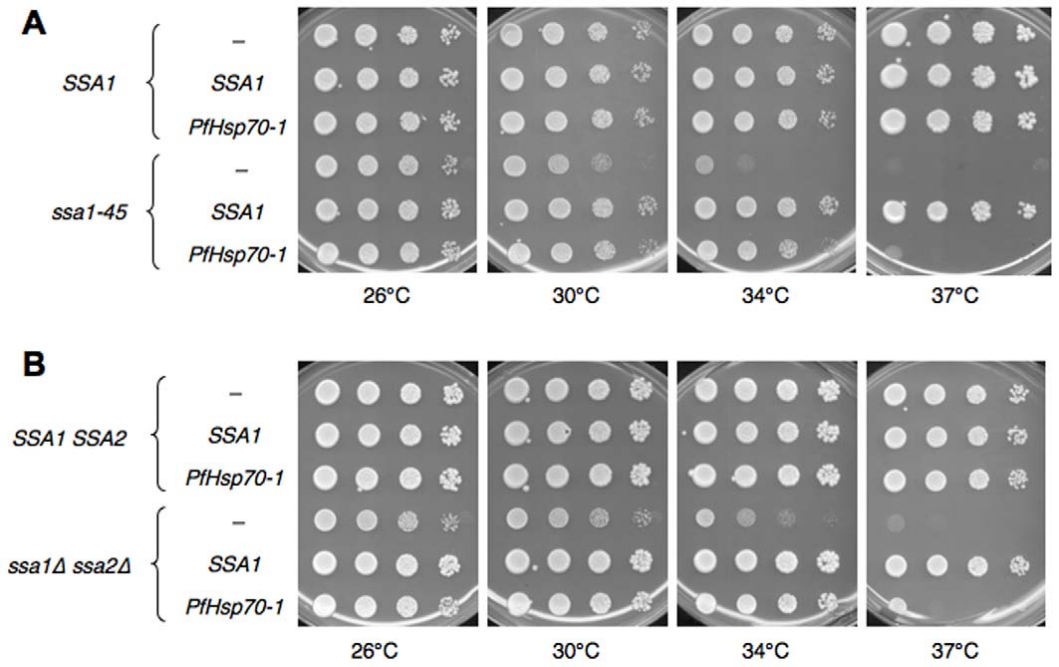
Expression of PfHsp70-1 in the *ssa1-45* and *ssa1Δssa2Δ* mutants partially complements the temperature sensitive phenotype and rescues the null phenotype. The indicated yeast strains were transformed with the designated plasmids, grown to log phase, and serially diluted on selective media and incubated at the noted temperatures for 2–3 d. The images shown are representative of at least three independent experiments, all demonstrating the same phenomena. “-” indicates an empty vector control.

Because Ssa1 and PfHsp70-1 are 71% identical (Figure 1), and because PfHsp70-1 is stable and soluble when expressed in yeast, we next examined whether PfHsp70-1 can rescue the thermosensitivity of *ssa1-45* yeast. The wild type and mutant strains were transformed with an “empty” vector and with the vectors engineered for Ssa1 or PfHsp70-1 expression. The transformants were serially diluted, plated on selective media, and grown at various temperatures. As shown in Figure 3A, the *SSA1* wild type strain grew robustly at all temperatures regardless of the introduced plasmid; therefore, PfHsp70-1 does not impart a negative effect on cell growth. In contrast, the *ssa1-45* strain with the empty vector grew poorly at elevated temperatures. As expected, however, when this mutant harbored a plasmid-borne wild type copy of *SSA1*, temperature sensitivity was rescued. When *ssa1-45* expressed PfHsp70-1, temperature sensitivity was rescued under all conditions except at the very highest temperature examined. These data indicate that PfHsp70-1 substitutes for Ssa1 function in yeast under most growth conditions.

The growth of *ssa1Δssa2Δ* yeast, along with the isogenic wild type strain, was also examined when the PfHsp70-1 or Ssa1 expression vectors or the empty vector control were introduced. The transformed wild type and mutant strains were again serially diluted, plated on selective media, and propagated at various temperatures. As shown in Figure 3B, the *ssa1Δssa2Δ* mutant transformed with an empty vector grew poorly at higher temperatures, but again, as expected, expression of wild type Ssa1 rescued temperature sensitivity. Expression of PfHsp70-1 also rescued growth at 34°C, but failed to rescue temperature sensitivity at 37°C. These data are consistent with the level of rescue observed in Figure 3A.

Because *P. falciparum* must thrive under oxidative conditions, and because chaperone function in cells is critical under these conditions (see Introduction), we next studied the ability of PfHsp70-1 to restore Ssa1-dependent growth in yeast exposed to oxidative stressors. The *ssa1Δssa2Δ* and the isogenic wild type strains transformed with the PfHsp70-1 and Ssa1 expression vectors and an empty vector were diluted and grown on media containing either cadmium chloride, copper sulfate, diamide, or hydrogen peroxide, each of which is known to induce an oxidative stress [62]. For example, cadmium and copper catalyze the formation of reactive oxygen species. Cadmium also causes a nonspecific stress in cells. In contrast, diamide oxidizes glutathione reductase, preventing the cell from eliminating reactive oxygen species, and hydrogen peroxide directly introduces reactive oxygen species in the cell.

**Figure 4:**
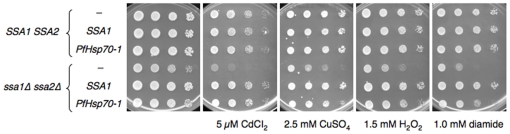
Expression of PfHsp70-1 in an *ssa1Δssa2Δ* mutant partially rescues sensitivity to oxidative stress. The indicated yeast strains transformed with the designated plasmids were grown to log phase, serially diluted, and plated on selective media in the absence of any additives or with the indicated amount of a stress-inducing agent. The plates were imaged after 2–3 d growth at 26°C. The images shown are representative of at least three independent experiments, all demonstrating the same phenomena. “-” indicates an empty vector control.

As shown in Figure 4, *ssa1Δssa2Δ* growth at 26°C is severely affected in the presence of each of these oxidative stressors; however, when *ssa1Δssa2Δ* cells express Ssa1, the growth defect is absent. Expression of PfHsp70-1 in *ssa1Δssa2Δ* yeast also rescued the growth defect on oxidative agents, although to a somewhat lesser extent than did the expression of Ssa1. Therefore, PfHsp70-1 at least partially supplants Ssa1 function during oxidative stress.

### PfHsp70-1 rescue requires conserved amino acid residues critical for chaperone function

In theory, the partial rescue observed upon PfHsp70-1 expression could have arisen from the expression of other complementing chaperones. For example, the expression of a foreign protein, such as PfHsp70-1, could induce a stress response, which in turn might induce the synthesis of Ssa1. To test this hypothesis, we undertook two lines of investigation. First, we generated PfHsp70-1 mutants that are predicted to disable Hsp70 function. One mutation, P434L, is the equivalent mutation found in *ssa1-45*, and resides in the substrate-binding domain of the Hsp70 [47]. The second mutation, G214D, is in the ATPase domain of the Hsp70 and inhibits ATP hydrolysis [41], thereby disabling the chaperone. These two mutated residues, marked with asterisks in Figure 1, are conserved from *E. coli* to mammals.

**Figure 5:**
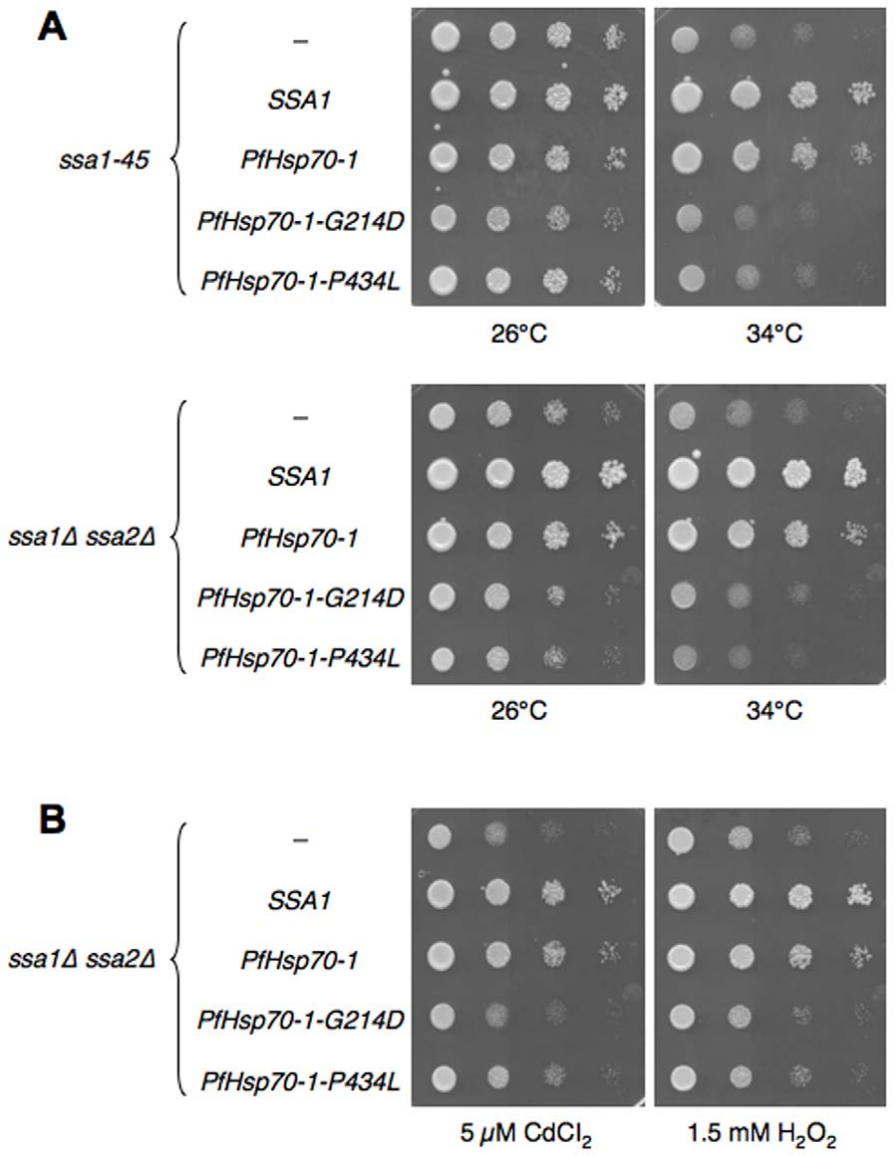
Mutations in the ATPase domain and in the SBD subvert the PfHsp70-1-mediated improvement of growth in the *ssa1-45* or *ssa1Δssa2Δ* mutants. (A) The indicated mutant strains transformed with the designated plasmids were grown to log phase, serially diluted, and plated on selective media and incubated at the indicated temperatures for 2–3 d. (B) Cells were plated on selective media containing the indicated stress-inducing agent, grown at 26°C, and imaged after 3 d. In both panels, the images shown are representative of at least three independent experiments, all demonstrating the same phenomena. “-” indicates an empty vector control.

When the *ssa1-45* and *ssa1Δssa2Δ* strains expressed these mutant forms of PfHsp70-1, we found that the strains remained thermosensitive (Figure 5A). In addition, the oxidative sensitivity of *ssa1Δssa2Δ* yeast was unaffected when the PfHsp70-1 mutants were expressed (Figure 5B). When these mutant forms of PfHsp70-1 were expressed in the wild type strains, no growth defect was observed, so the PfHsp70-1 mutants do not negatively affect growth (data not shown).

**Figure 6:**
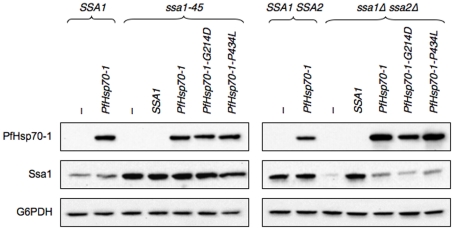
Ssa1 is not upregulated in response to wild type or mutant PfHsp70-1 expression. The indicated yeast strains transformed with the indicated plasmids were grown in liquid culture to log phase at 26°C and were then incubated at 37°C for 30 min. Equivalent amounts of cells were harvested, and samples were processed as described in the “Methods”. Note: The anti-Ssa1 antiserum crossreacts with Ssa3 and/or Ssa4, since there is a faint band in the *ssa1Δssa2Δ* strain transformed with the empty vector. This antiserum recognizes the last 56 amino acids in Ssa1 (E. Craig, personal communication). Also note that the levels of the PfHsp70-1 mutants are similar to the level of the wild type protein (compare the last three lanes, top-right panel). The absence of an increased level of Ssa1 in *ssa1-45* yeast containing the *SSA1* expression vector most likely results from the fact that the protein binds to its message and represses translation [71]. Thus, the expression of Ssa1 is expected to depress both its own synthesis and the synthesis of the Ssa1-45 mutant protein.

As a second approach to establish that the rescue conferred by PfHsp70-1 expression was specific, we measured the levels of Ssa1 in each of the examined strains. As shown in Figure 6, Ssa1 is not upregulated in a PfHsp70-1-dependent manner. Together, these data strongly suggest that PfHsp70-1 specifically rescues growth defects associated with *ssa1* mutant alleles.

### PfHsp70-1 also repairs cellular events that are compromised in *ssa1* mutant yeast

**Figure 7:**
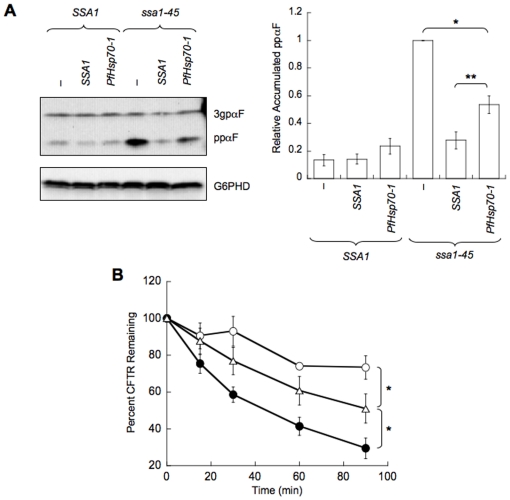
PfHsp70-1 partially restores translocation and ERAD efficiencies in the *ssa1-45* mutant. (A) The indicated transformed strains were grown to log phase at 26°C, and were then incubated at 37°C for 15 min. Equal amounts of cells were harvested and processed as described in the “Methods”. The graph shows the relative accumulation of ppαF. The accumulation of ppαF in the *ssa1-45* strain transformed with an empty vector was set to 1 (i.e., maximal accumulation of ppαF). Error bars indicate SEM, n = 4 independent experiments, and statistical significance was calculated using a Student's t-test; * denotes p<0.05, and ** denotes p<0.005. (B) ERAD was examined using cycloheximide chase analyses as described in the “Methods”. The percent CFTR remaining over time was calculated for *ssa1-45* transformed with an empty vector (○), an Ssa1 expression vector (•), or a PfHsp70-1 expression vector (▵). CFTR degradation in the wild type *SSA1* strain was identical to that of *ssa1-45* transformed with the Ssa1 expression vector (data not shown). Error bars depict the SEM, n≥4, and statistical significance was calculated using a Student's t-test; * denotes p<0.05.

As described in the Introduction, Ssa1 directly catalyzes various processes, including protein translocation into the ER and the ER associated degradation (ERAD) of misfolded membrane proteins. Previous studies that uncovered these phenomena utilized the *ssa1-45* strain [46], [47], [63]. Therefore, we measured the ability of PfHsp70-1 to repair protein translocation and ERAD defects in the *ssa1-45* mutant and compared the effects when this strain contained either a vector control or an exogenous copy of *SSA1*. First, we discovered that the expression of PfHsp70-1 significantly decreased the amount of untranslocated ppαF that accumulated in *ssa1-45* yeast (Figure 7A). Second, we found that the degradation of CFTR, an integral membrane protein that serves as an ERAD substrate in yeast [46], was accelerated upon the expression of PfHsp70-1 in the *ssa1-45* strain (Figure 7B). Together, these data indicate that PfHsp70-1 repairs specific Hsp70-dependent activities that are defective in *ssa1* yeast.

## Discussion

The purpose of this study was to design a yeast-based expression system to characterize the function of the *P. falciparum* Hsp70, PfHsp70-1. To this end, expression plasmids were constructed in which the endogenous promoter for the yeast cytosolic Hsp70, Ssa1, controlled the production of PfHsp70-1. In parallel, an Ssa1 expression vector under the control of its own promoter was used. Our combined data indicate that PfHsp70-1 partially restores several well-characterized Hsp70-mediated activities in *ssa1* mutant cells. More generally, our results indicate that the function of PfHsp70-1 can be studied in a new, genetically tractable eukaryotic system, and suggest strongly that PfHsp70-1 performs housekeeping chaperone functions in the malarial parasite, *P. falciparum*.

It is important to place our findings in the context of a previous study in which the ability of PfHsp70-1 to replace the *E. coli* Hsp70, DnaK, was examined [64]. It was found that high-level expression of PfHsp70-1 rescued the temperature sensitive growth phenotype of strains with mutations in DnaK, but PfHsp70-1 failed to restore growth in a DnaK deleted strain at high temperature. Cellular functions associated with DnaK activity were not examined. However, by constructing DnaK-PfHsp70-1 chimeras, Blatch and colleagues discovered that the SBD of PfHsp70-1 was critical for improved thermotolerance. This result suggests that the range of peptides bound by an Hsp70 chaperone is important to maintain viability. In our study, we again examined the ability of an evolutionary distant chaperone to function in a genetic system. But, we purposely chose to employ a genetically amenable eukaryote so that compartment-specific phenotypes could be assessed (i.e., ERAD and translocation), as well as more general responses (i.e., thermosensitivity and oxidative stress).

Does *P. falciparum* Hsp70 represent a potential target to identify novel antimalarial agents, and might our yeast expression system be employed to identify these compounds? Yeast have long been used for high throughput screening efforts [65]. However, it is not clear whether potent inhibitors could be identified that selectively block the action of PfHsp70-1 but leave the human host's Hsp70 untouched. Some progress toward this goal has been achieved [66], [67]. Nevertheless, even if an Hsp70 inhibitor compromised the function of both human and *P. falciparum* Hsp70, examples are known in which modest depletion of Hsp70 inhibits the growth of an unwanted cell-type, such as a cancer cell, but has no effect on normal cells [68]. In addition, the partial inhibition of another chaperone, Hsp90, has been shown to sensitize pathogenic yeast to antibiotics [69]. Therefore, we suggest that partial inhibition of Hsp70 may potentiate the effects of established antimalarial drugs. This effect might be particularly robust during fever, when the levels of *P. falciparum* Hsp70 and Hsp40 rise in order to maintain parasite homeostasis [15], [38], [67]. In fact, a compound that targets both Hsp70 and Hsp90 has been shown to inhibit protein trafficking and the growth of *P. falciparum* in red blood cells [25]–[27].

Based on the development of this new system, we can now examine the functions of other cytosolic Hsp70s from *P. falciparum* in *ssa1* yeast. Moreover, we can examine the function of parasitic Hsp70s that reside in organelles, for example the ER or mitochondria, in yeast strains lacking functional versions of the analogous organellar chaperone(s). Given the large number of Hsp70 homologs in the malarial parasite, and near absence of information on their activities, we are confident that these efforts will prove worthwhile in future efforts.
